# Is the Transport of a Gadolinium-Based Contrast Agent Decreased in a Degenerated or Aged Disc? A Post Contrast MRI Study

**DOI:** 10.1371/journal.pone.0076697

**Published:** 2013-10-11

**Authors:** Marta Tibiletti, Fabio Galbusera, Cristina Ciavarro, Marco Brayda-Bruno

**Affiliations:** 1 Laboratory of Biological Structure Mechanics, IRCCS Galeazzi Orthopaedic Institute, Milan, Italy; 2 Department of Internal Medicine II-Cardiology, Ulm University, Ulm, Germany; 3 Department of Spine Surgery III, IRCCS Galeazzi Orthopaedic Institute, Milan, Italy; University Medical Center (UMC) Utrecht, The Netherlands

## Abstract

A post contrast magnetic resonance imaging study has been performed in a wide population of low back pain patients to investigate which radiological and phenotypic characteristics influence the penetration of the contrast agent in lumbar discs in vivo. 37 patients affected by different pathologies (disc herniation, spondylolisthesis, foraminal stenosis, central canal stenosis) were enrolled in the study. The selected population included 26 male and 11 female subjects, with a mean age of 42.4±9.3 years (range 18–60). Magnetic resonance images of the lumbar spine were obtained with a 1.5 T scanner (Avanto, Siemens, Erlangen, Germany) with a phased-array back coil. A paramagnetic non–ionic contrast agent was injected with a dose of 0.4 ml/kg. T1-weighted magnetic resonance images were subsequently acquired at 5 time points, 5 and 10 minutes, 2, 4 and 6 hours after injection. Endplates presented clear enhancement already 5 minutes after injection, and showed an increase in the next 2 hours followed by a decrease. At 5 and 10 minutes, virtually no contrast medium was present inside the intervertebral disc; afterwards, enhancement significantly increased. Highly degenerated discs showed higher enhancement in comparison with low and medium degenerated discs. Discs classified as Pfirrmann 5 showed a statistically significant higher enhancement than Pfirrmann 1, 2 and 3 at all time points but the first one, possibly due to vascularization. Disc height collapse and Modic changes significantly increased enhancement. Presence of endplate defects did not show any significant influence on post contrast enhancement, but the lack of a clear classification of endplate defects as seen on magnetic resonance scans may be shadowing some effects. In conclusion, disc height, high level of degeneration and presence of Modic changes are factors which increase post contrast enhancement in the intervertebral disc. The effect of age could not be demonstrated.

## Introduction

The intervertebral disc (IVD) is the largest avascular structure in the human body. A sparse population of cells produces and maintains the extracellular matrix, which confers to the healthy IVD the ability to withstand the compressive loads, and to allow spine bending and twisting [1]. Disc cells require an adequate nutrient exchange to survive and be viable [2], mostly from the capillaries of the subchondral plate of the vertebral body, across the layer of hyaline cartilage that constitutes the endplate [3]. Once the molecules of the nutrient (glucose and oxygen) have reached the disc matrix, they move towards the core by diffusion, thanks to gradients established by cellular metabolic rate [4], [5]. At the same time, lactic acid produced by cell anaerobic metabolism diffuses out of the disc, since its accumulation would consequence in a pH decrease and reduced cell viability [6]. An alteration of this nutrient pathway is considered as one of the possible causes of IVD degeneration [2]. One mechanism may be the reduction of cartilage permeability via calcification [7], [8]; alternatively, subchondral bone sclerosis may result in fewer and smaller pores through which transport can occur. Cadaveric tests allow for the calculation of endplate porosity and permeability. Benneker et al [9] quantified pores in the osseous endplate and reported a decrease in the density of holes (range, 20–50 µm) with increasing degeneration and decreasing glycosaminoglycan content. However, a recent works by Rodriguez et al [10] reported that total endplate permeability and porosity increase with ageing and degeneration.

The direct study of nutrient exchange is mostly performed *in vivo*, as *in vitro* investigations are complicated by blood clogging in the vascular buds of the endplate [11]. Data regarding human IVD can be obtained either through needle sensors or imaging methods. Needle sensors measuring oxygen pressure in IVD have been used in a canine model [12] and in human patients during surgery [13], and proved that partial pressure of oxygen is low at disc centre, but no correlation with the degeneration degree could be shown. One study showed reduced transport of nitrous oxide in patients with neuromuscular scoliosis particularly at the curve apex [14].

Imaging techniques require the injection of a tracer in the blood circulation and the study of its penetration into the IVD over time. Post contrast MRI studies have been introduced first in animal models [15]–[17], and then translated to human subjects by Rajasekaran et al [18]–[20]. In these studies, a paramagnetic non-ionic contrast agent is injected intravenously and subsequent T1-weighted MRIs are taken at different time points: over the course of hours, it is possible to see the contrast agent being transported from the endplate to the disc centre. Qualitative and semi-quantitative analyses allow studying the transport properties of the contrast agent through the endplate. Rajasekaran et al concluded that discs with mild degeneration have reduced transport, while highly degenerated disc a markedly increased one [18]. Later Niinimaki et al [21] using a similar technique showed that post-contrast enhancement in the disc centre was indeed increased in highly degenerated discs, but no decrease was found in mildly degenerated ones. Therefore, despite the potential of post contrast MRI to investigate disc nutrition, the few available studies could not generally confirm the hypothesis of calcification and reduction of permeability of the vertebral endplates as a trigger for reduced nutrition of the disc cells and subsequent initiation of disc degeneration. Furthermore, previous studies did not clearly assess the role of factors such as disc height and presence of Modic changes (MCs), the relevance of which in determining post contrast enhancement should be expected to be significant.

Given these conflicting and not comprehensive results present in the scientific literature, a post contrast MRI study has been performed in a wide population of low back pain patients to investigate which morphological, radiological and anamnestic characteristics including age and degeneration influence the penetration of contrast agent in lumbar discs *in vivo* as a model for nutrition of the disc cells.

## Materials and Methods

### Ethics Statement

The protocol was approved by the appropriate Ethical Committee (ASL Città di Milano, Milan, Italy). Written informed consent was obtained from all patients.

### Patient Population

37 patients referred to our hospital with complaints of low back pain were enrolled in the study. These patients were affected by different pathologies (disc herniation, spondylolisthesis, foraminal stenosis, central canal stenosis). Patients were chosen among the ones referred for contrast-enhanced MRI at the lumbar spine in our institution and were willing to undergo the relatively long protocol required by the study. The selected population included 26 male and 11 female subjects, with a mean age of 42.4±9.3 years (age range 18–60). Each patient received detailed information regarding the study protocol and gave her/his consent. Exclusion criteria were age less than 18 and higher than 60, contrast agent allergy, reduced renal functionality and impossibility of undergoing MR acquisition.

### MRI Protocol

Magnetic resonance images (MRI) of the lumbar spine were obtained with a 1.5 T scanner (Avanto, Siemens, Erlangen, Germany) with a phased-array back coil. Firstly standard MRI examination included routine T1-weighted sagittal (TR/TE = 500/13 ms, 13 slices) and axial (TR/TE = 550/10 ms, 29 slices) acquisition and T2-weighted turbo spin echo sequences with sagittal orientation (TR/TE = 4180/104 ms, 13 slices). In addition, a paramagnetic non–ionic contrast agent (proHance®, gadoteridol, Bracco Diagnostics, Princeton, NJ, USA), was injected with a dose of 0.4 ml/kg (a double dose with respect to normal clinical applications), followed by a 10 ml bolus of saline. The set of images analysed in the paper were T1- weighted MR sagittal images (TR/TE = 500/13 ms, Echo Train Length = 3, FA = 150°, Average = 1, 7 slices, thickness = 4 mm, number of pixels = 384×384, pixel spacing = 0.83×0.83 mm) and were acquired before contrast agent injection and at 5 subsequent time points, 5 minutes, 10 minutes, 2 hours, 4 hours and 6 hours after injection (respectively PRE, POST_5MIN, POST_10MIN, POST_2H, POST_ 4H, POST_6H). A radiologist graded the degeneration of each disc from T12/L1 to L5/S1, according to the Pfirrmann scale [22], and noted the presence and type of MCs and endplate defects. The Pfirrmann scale is based on the nucleus pulposus (NP) signal intensity in T2-weighted images and disc height, and divides discs into 5 categories, from 1 being a healthy disc with homogeneous, bright white NP and normal height till 5, being a black, collapsed, severely degenerated disc. MCs are common variations of signal intensity in the endplate and vertebral body seen on magnetic resonance images. MCs are classified into type I (regarded as signs of an ongoing inflammatory condition characterised by edema), type II (regarded as signs of fatty marrow degeneration) and type III (regarded as signs of sclerosis). Endplates presenting different types of MCs are also frequent.

### Image Analysis

For each subject, one slice corresponding to the sagittal centre of the vertebra was chosen among the slices of T1-weighted pre-contrast images. The corresponding slices of the T2 weighted pre-contrast scan and T1 post-contrast scans (POST_5MIN, POST_10MIN, POST_2H, POST_ 4H, POST_6H) were selected, and each of them was registered to ensure alignment with respect to PRE slice using Elastix, a registration toolkit based on the National Library of Medicine Insight Segmentation and Registration Toolkit (ITK) [23]. All the subsequent evaluations were based on these images. The six T1-weighted images per subject were used to infer the change in T1 relaxation time, as paramagnetic contrast agents act changing this value in the tissues where it pools. The T2-weighted image, one per subject, were used to evaluate NP brightness, which is a function of the level of hydration and therefore of degeneration in the disc [24].

**Figure 1:**
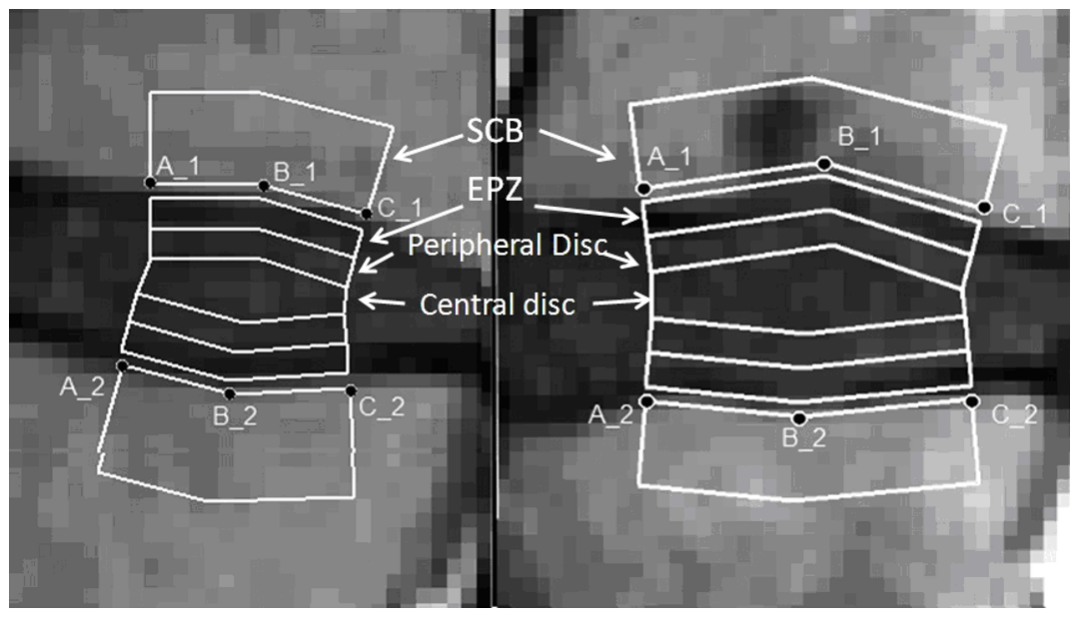
Example of the segmentation of discs in Regions of Interest. The disc on the left present a smooth, defectless endplate: all the ROIs were studied in this case. The disc on the right present a clear Schmorl’s node: only the central disc values were considered. An operator manually places points (A, B, C) to delimitate the border between endplate and disc. Care was taken so that the line between A_1 and A_2 and the line between C_1 and C_2 draw a line perpendicular to the endplates. The mean value of such distances was used to approximate the height of the disc. Each disc was then automatically divided by the software into 5 layered ROIs, named cranial and caudal Endplate Zone (EPZ), cranial and caudal Peripheral Disc and Central Disc. The EPZ is the ROI closer to the vertebral bone, and its height is 1/5 of the disc height; the Peripheral Disc borders with the EPZ and its height is 1/5 of the disc height, and the Central Disc is defined as the region in between the upper and lower Peripheral disc ROIs. A ROI corresponding to the vertebral body close to the disc was also defined starting from the border point with a fixed, arbitrary thickness of 6 pixel = 4.98 mm (Subchondral Bone, SCB): a thin layer of 1 pixel height separate SCB and EPZ, as it normally represent the “grey” border between bone and disc.

In order to study the transport of the contrast medium in the IVD, it is necessary to define region of interest (ROI) to segment the disc. Purpose software was written to analyze images using Matlab® (MathWorks, Natick, Massachusetts USA). Firstly an operator (M.T.) selected 3 points that define the border between the disc and the vertebra for each endplate. Each disc was then automatically divided by the software into 5 layered ROIs, named upper and lower endplate zone (EPZ), upper and lower Peripheral Disc and Central Disc. A ROI corresponding to the vertebral body close to the disc was also defined and called Subchondral Bone (SCB). For a detailed description of the algorithm, see the caption of Figure 1.

Cases of extremely degenerated discs (height< = 3 mm) and severe spondylolisthesis were excluded. For all the considered discs, the values of the Central Disc ROI were studied.

The profile of the enhancement of the pixel brightness due to the presence of contrast agent over time has been studied only in discs having a smooth, defectless endplate.

In order to quantitatively study the transport of the contrast agent, the mean values of pixels inside each of the selected ROI was calculated for each of the six time-points. The values obtained for the post-injection scans were subtracted with the correspondent value in the pre-contrast scan, to define the enhancement:
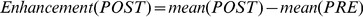
(1)


The same segmentation was applied to the T2-weighted (registered) scan; also a 3×5 rectangular pixel region was manually place in the Cerebral Spinal Fluid (CSF) close to the disc as reference.

The index used in this case was CSF-adjusted T2-weighted signal intensity (CSF-adj T2-weighted SI), calculated as the mean value of the pixel in Central and Peripheric disc ROIs, divided for the mean value of the pixel in the CSF ROI [24], [25].

### Statistical Analysis

In order to determine the evolution of enhancement over time and across different ROIs, Rank Sum Test was used. In case the analysis involved 3 groups, Kruskal-Wallis One Way Analysis of Variance, was executed, followed by a Multiple Comparison Procedures with Dunn's Method.

In order to determine if different level of degeneration as identified by 5-levels Pfirrmann Score have an influence on Enhancement in Central Disc, Kruskal-Wallis One Way Analysis of Variance was carried out, followed by Multiple Comparison Procedures.

To test if CSF-adj T2-weighted SI values vary among discs with different Pfirrmann score, Kruskal-Wallis One Way Analyses of Variance, followed by a Multiple Comparison Procedure, were carried out.

In order to determine the presence of correlations between CSF-adj T2-weighted SI and enhancement at Central Disc, age and disc height, Spearman rank order correlation was used. The same statistical tool was used for investigating the correlation between age and disc height and enhancement at Central Disc.

To test if the presence of MCs in the endplate influences the Enhancement in the Central Disc, Rank Sum Test was carried out in case of segmentation of the population in two groups (disc with adjacent endplates with at least a MC and without any MC); in case the population was divided in more than two groups Kruskal-Wallis One Way Analysis of Variance, followed by a Multiple Comparison Procedure, were carried out.

To test if the presence of at least one endplate defect in the endplate influences the Enhancement in the Central Disc with respect to the disc having no endplate defect, Rank Sum Test was carried out for the two groups. The same test was use to discern significant differences between disc with “irregular” endplate and all other disc.

In order to discriminate the relative importance of different variables on the enhancement, a Univariate analysis of variance was carried out.

Non-parametric tests were chosen as a Shapiro-Wilk test confirmed a non- normal distribution of data in all cases. Presence of correlation between data was studied by Spearman rank order correlation. Differences were considered significant when p<0.05.

## Results

### Radiological Analysis

The radiological evaluation of the 37 subjects graded a total of 222 levels, from T12-L1 to L5-S1. Among these, 8 levels were excluded from further analysis, 2 due to severe anterolisthesis (both at L5/S1) and 6 because too thin to be segmented (1 L2/3, 1 L4/5, 4 L5/S1). The remaining 214 discs were graded using Pfirrmann classification as follows: 50 grade 1, 43 grade 2, 58 grade 3, 48 grade 4, 15 grade 5.

Regarding the endplates, 50 upper endplates (UEPs) and 70 lower endplates (LEPs) presented Schmorl’s nodes. 39 levels presented a Schmorl’s node at both endplates, 42 levels at one. Also, 11 discs were noted by the radiologist as having at least one *“irregular endplate”*.

MCs were present on 46 UEPs (15 MC I, 27 MC II, 4 MC I/II) and 45 LEPs (12 MC I, 29 MC II, 4 MC I/II). 36 levels present MCs at both endplates, 19 only at one. In order to simplify the statistical analysis, it was preferred to group together as MC type I/II all disc presenting at least one MC type I/II and disc presenting at different endplates one MCs type I and one type II.

### Time Curve

**Figure 2:**
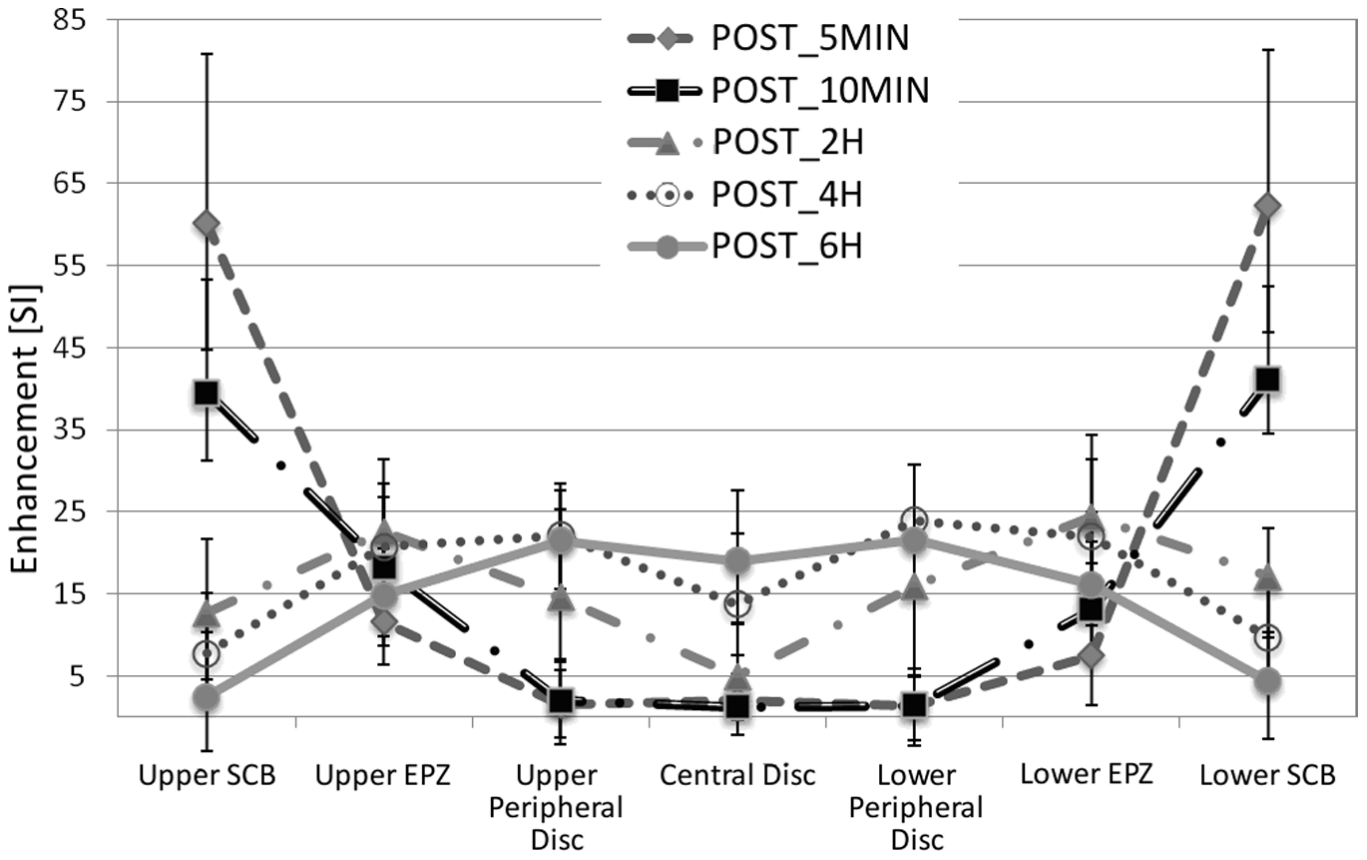
Representation of Median and Percentiles (25% and 75%) post contrast enhancement in all ROIs in 107 disc, from the upper SCB on extreme left to the lower SCB on the extreme right, at all time points considered.

**Figure 3:**
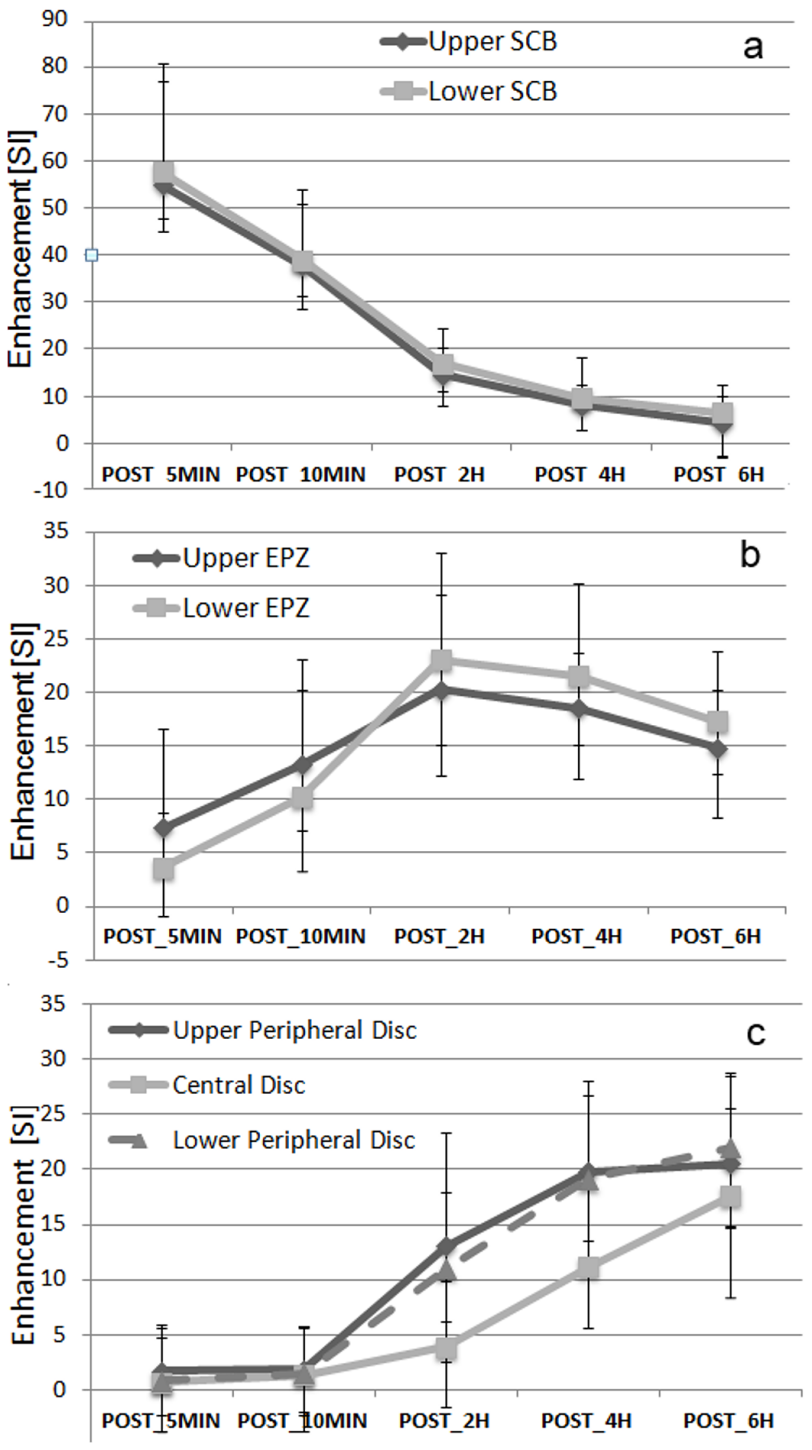
Alternative representation of data presented in Figure 2. In order to better appreciate the evolution of Enhancement (Median and 25% and 75% Percentiles) over time-points, (a) represents Lower and Upper SCB, (b) the Upper EPZ and Lower EPZ, (c) the Upper and Lower Peripheral Disc and Central disc.

Figure 2 represents the median ± quartiles of each ROI at each time-point. This analysis has been restricted to 107 discs presenting no evident defect or MCs at the endplates. The same data are also reported in an alternative way in Figure 3, where it is possible to appreciate the evolution of Enhancement over time for each ROI.

Values at SCB peaked at the first time point after injection and decreased thereafter. Enhancement at each time point was lower than the previous one both for upper and lower SCB (p<0.001), with values just above zero at last time point.

EPZs presented clear enhancement already at POST_5MIN, and showed an increase between POST_5MIN and POST_10MIN, (Upper EPZ: p = 0.002, Lower EPZ: p<0.001), and between POST_10MIN and POST_2H (Upper EPZ: p = 0.005, Lower EPZ: p<0.001); there was a slight decrease between POST_2H and POST_4H, which was not significant for upper (p = 0.118) and significant for lower EPZ (p = 0.027). A statistically significant decrease between POST_4H and POST_6H (p<0.001) was present at both sides. EPZ had lower values than SCB at POST_5MIN and POST_10MIN, but at POST_2H, POST_4H, POST_6H they showed a higher enhancement that SCB (p<0.001 at both sides, at all time points). Enhancement at EPZs was also significantly higher than at Peripheral Discs at POST_5MIN, POST_10MIN and POST_2H (at both sides, p<0.001), not different at POST_4H (upper EPZ: p = 0.483, lower EPZ: p = 0.809), while at POST_6H Peripheral Discs were higher than EPZs (upper EPZ: p<0.001, lower EPZ: p = 0.002).

At POST_5MIN and POST_10MIN, virtually no contrast medium was present at Central and Peripheral disc ROIs. At POST_2H, enhancement at Peripheral and Central discs significantly increased with respect to the previous time point (p<0.001); values at Central disc were significantly lower than at Peripheral discs (p<0.001, multiple comparison Central vs each Peripheral disc p<0.05). Similar observations can be made at POST_4H: enhancement at Peripheral and Central discs significantly increased with respect to POST_2H (all cases, p<0.001), but at Peripheral disc ROIs were significantly higher than at Central disc. At POST_6H, Central disc presented a slight, non significant lower enhancement than Peripheral disc (p = 0.092).

### Degeneration as Classified by Pfirrmann Score

**Figure 4:**
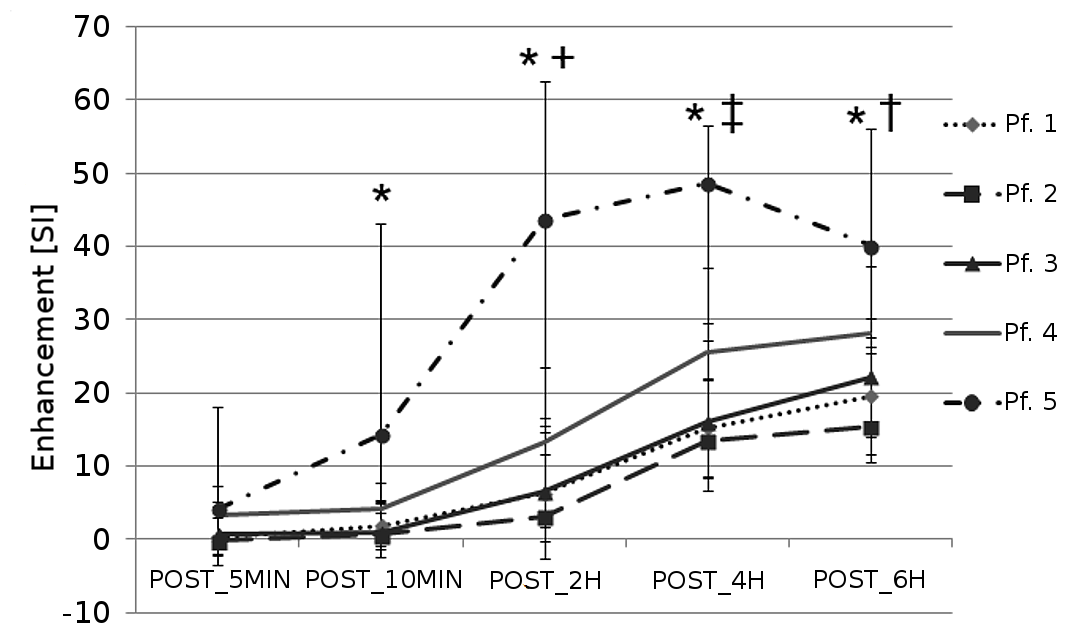
Post-contrast enhancement in Central Disc in disc divided on level of degeneration following Pfirrmann grading. * indicates that Pfirrmann 5 discs enhance statistically more that Pfirrmann 1, 2 and 3,+indicates that Pfirrmann 4 enhance more than Pfirrmann 1,2 and 3 discs, ‡ indicates that Pfirrmann 4 enhances more than Pfirrmann 1 and 2, † indicates than Pfirrmann 4 enhances more than Pfirrmann 2.

Figure 4 shows the time curve of the Enhancement for the different Pfirrmann Scores. In general, highly degenerated discs showed higher enhancement in comparison with low and medium degenerated discs. At all time points, the Analysis of Variance shows a statistically significant difference among groups (at POST_5MIN p = 0.012, all other time points p<0.001). The Multiple Comparison Procedure showed that discs classified as Pfirrmann 5 have significant higher enhancement than Pfirrmann 1, 2 and 3 at all time points but POST_5MIN. Moreover, Pfirrmann classified as Pfirrmann 4 enhanced statistically more than Pfirrmann 1 (at POST_2H and POST_4H), than Pfirrmann 2 (POST_2H, POST_4H, POST_6H) and Pfirrmann 3 (POST_2H).

### Degeneration as Evaluated by the CSF-adjusted T2-weighted SI

**Figure 5:**
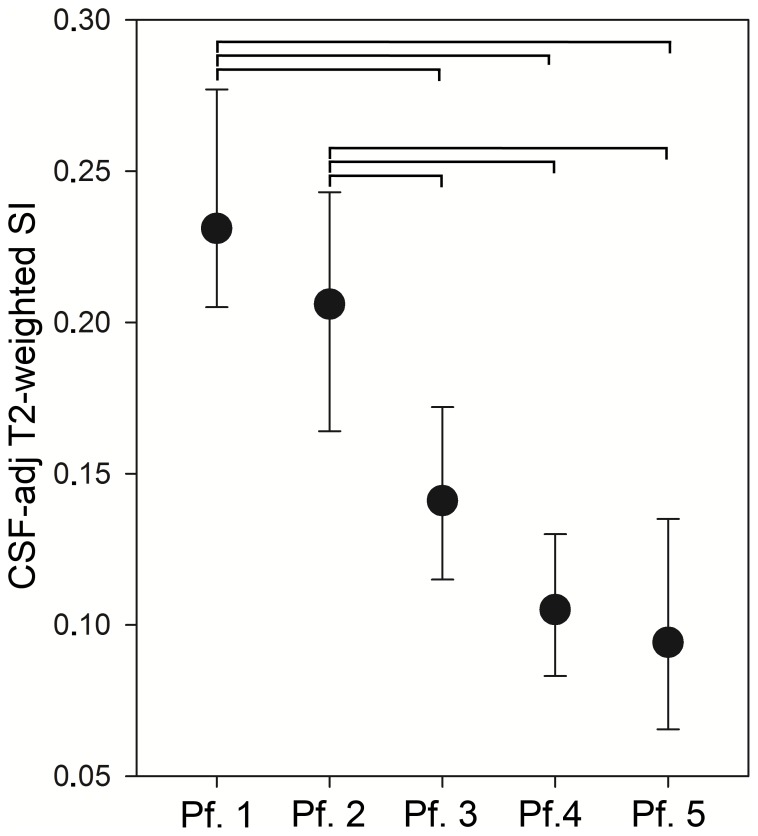
Median CSF- adjusted T2-weighted Signal Intensity in Central and Peripheral disc in disc divided on Pfirrmann grading. Error bars indicates 25% and 75% percentiles. Horizontal bars indicate which pairs have significant different values.

Figure 5 shows the median value of CSF-adj T2-weighted SI per each Pfirrmann score. CSF-adj T2-weighted SI values resulted higher in less degenerated disc: both Pfirrmann 1 and 2 demonstrated an higher Signal Intensity than Pfirrmann 3, Pfirrmann 4 and Pfirrmann 5 (p-value for Multiple Comparison, p<0.05).

### Correlation of T2-weighted CSF-adjusted SI with Enhancement at Central Disc

**Figure 6:**
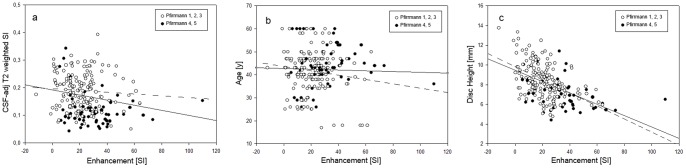
Scatter plots of the Enhancement versus parameters expected to have an influence on the transport of the contrast agent. (a) CSF-adj T2-weighted versus Enhancement for POST_6H, with the linear fitting line for all data (solid line, R = −0.219, p<0.001) and only Pfirrmann 1,2 and 3 discs (dashed line, R = −0.06, p = 0.45). (b) Subject's age versus Enhancement for POST_6H, with the linear fitting line for all data (solid line, R = −0.219, p = 0.001) and only Pfirrmann 1,2 and 3 discs (dashed line, R = −0.014, p = 0.86). (c) Disc height versus Enhancement for POST_6H, with the linear fitting line for all data (solid line, R = −0.512, p<0.001) and only Pfirrmann 1,2 and 3 discs (dashed line, R = −0.469, p<0.001).

Correlation between CSF-adj T2-weighted SI and enhancement at Central Disc, subjects age and disc height was investigated at all time points, both considering all discs and only for Pfirrmann 1,2 and 3 discs (i.e. disc presenting low to medium degeneration). For all discs, significant negative correlation (p<0.001) between CSF-adj T2-weighted SI and enhancement was found for POST_2H (R = −0.247), POST_4H (R = −0.305), POST_6H (R = −0.219, Fig. 6a) a negative correlation (p<0.05) was also observed between T2-weighted CSF-adj SI and age (R = −0.159, p = 0.0204), whereas a positive correlation was found between T2-weighted CSF-adj SI and disc height (R = 0.198, p = 0.0036). Considering only enhancement in Pfirrmann 1, 2 and 3 discs, no statistically significant correlations were found.

### Correlation between Enhancement at Central Disc and Age

There was no significant correlation between age and enhancement at Central Disc at any time point, nor considering all discs, nor considering low to medium degenerated disc (Fig. 6b).

### Correlation between Enhancement at Central Disc and Disc Height

Correlation between disc height and enhancement at the Central Disc at all time points for all discs and only Pfirrmann 1, 2 and 3 discs was investigated. Considering all discs, a negative statistically significant correlation was present at all time points, with the exclusion of POST_5MIN (POST_10MIN: R = −0.184, P = 0.007; POST_2H: R = −0.486, P<0.0001; POST_4H: R = −0.535, P<0.0001; POST_6H: R = −0.514, P<0.0001). For Pfirrmann 1, 2 and 3 discs, a significant correlation is present at POST_2H (R = −0.394), POST_4H (R = −0.459), POST_6H (R = −0.4690), in all case p<0.0001.

### Presence of Modic Changes

The subgroup of discs having at least one endplate with MCs (n = 55) reported significantly higher contrast enhancement than the rest of the discs (n = 159), at all time points but the first (POST_5MIN: p = 0.284; POST_10MIN: p = 0.007; POST_2H: p<0.001; POST_4H: p<0.001; POST_6H: p<0.001).

If the type of MC was used to define subgroups, Kruskal-Wallis One Way Analysis of Variance showed the existence of differences among the different types of MC at all time points, with the exclusion of POST_5MIN (POST_5MIN: p = 0.443; POST_10MIN: p = 0.030; POST_2H: P<0.001, POST_4H: p<0.001; POST_6H: p = 0.001). A Multiple Comparison Procedure showed furthermore that at POST_10MIN, MCs II had significantly higher enhancement than disc with no MC. Moreover, at POST_2H, MCs II and MCI/II were each significantly higher than MCs I and MCs 0. At POST_4H, MC I/II were higher than MC I and MCII, while MCII were higher than MC0. At POST_6H, no significant difference was found, even though MC I/II were higher than all others and MC I reached similar values than MC 0.

### Presence of Endplate Defects

A significant difference between enhancement in Central Disc between disc adjacent to at least one endplate defect and disc adjacent to defectless endplate was not reached at any time-point (POST_5MIN P = 0.448 POST_10MIN: P = 0.471; POST_2H: P = 0.110; POST_4H: P = 0.099; POST_6H: P = 0.075).

Rank Sum Test demonstrated that the 11 discs having “irregular” endplates did enhance strongly more than the others from POST_10MIN on (POST_5MIN: p = 0.129; POST_10MIN: P = 0.001; POST_2H: P<0.001; POST_4H: p<0.001; POST_6H: P = 0.019).

Since “irregular” endplates are adjacent only to discs with advanced degeneration, these discs have been compared with the other “severely degenerated” (Pfirrmann 4 and 5, for a total of 52 discs), and a significant difference was found at POST_10MIN and POST_2H (POST_5MIN: p = 0.568; POST_10MIN: p = 0.038; POST_2H: p = 0.030; POST_4H: p = 0.140; POST_6H: p = 0.420).

### Multivariate Analysis

Table 1 shows the results of with different variables considered for creating a multivariate model for enhancement at POST_2H, POST_4H, POST_6H. Disc height was a statistical significant variables in all the considered models (p<0.0001). The degeneration grade was the second strongest predictor for the enhancement at all time points. The CSF-adj T2-weighted SI was less markedly associated with the enhancement, with statistical significance only at POST-4H. The presence of MCs was the third determinant factor at POST_2H and POST_4H, but not at the last time point.

**Table 1:** The significant determinants of central disc enhancement: multivariate associations.

Univariate analysis of variance (all covariance)							
	POST_2H		POST_4H		POST_6H		
Variables	F	p	F	p	F	p	
Disc height	45.055	**<0.0001**	61.961	**<0.0001**	55.631	**<0.0001**	
Presence of EP Defect	2.201	0.139	3.019	0.084	0.981	0.323	
Presence of MC	7.861	**0.006**	5.158	**0.024**	2.007	0.158	
Pfirrmann grade	9.886	**0.002**	10.867	**0.001**	7.741	**0.006**	
**Variables**							
Disc height	46.415	**<0.0001**	62.544	**<0.0001**	57.108	**<0.0001**	
Presence of EP Defect	1.92	0.167	2.667	0.104	0.834	0.362	
Presence of MCs	18.032	**<0.0001**	12.572	**<0.0001**	6.994	**0.009**	
CSF-adj Tt2-weighted SI	2.023	0.156	5.083	**0.025**	1.389	0.24	
**Variables**							
Disc height	36.625	**<0.0001**	56.992	**<0.0001**	56.337	**<0.0001**	
Presence of Irregular Endplate	6.722	**0.01**	0.067	0.796	1.157	0.283	
Presence of MCs	3.691	**0.056**	4.054	**0.045**	2.255	0.135	
Pfirrmann grade	6.907	**0.009**	10.463	**0.001**	8.493	**0.004**	

Endplate defects were not a significant factor for the discs with Schmorl’s nodes. This factor was significant for “irregular” endplates, but only at POST_2H.

### Noise

Data regarding the enhancement at Central Disc in Pfirrmann 1 and 2 discs (n = 91) at POST_5MIN can be considered as pure noise, as virtually no contrast agent should have reached the central part of the disc in such a short time, this data could. Data presented a mean value of 1.2, and One sample t-test found that these values were not significantly different with respect to a distribution having mean equal to zero. However, a high standard deviation (8.1) was found. For reference, the mean value in central disc at POST_6H is 19 and the mean value of PRE in the same ROIs was 99 (standard deviation 13).

## Discussion

In this work, the transport through the endplates and into the intervertebral disc of a paramagnetic non-ionic contrast agent over time was studied with serial post contrast MRI. Disc height, high level of degeneration and presence of MCs increased post contrast enhancement in the centre of intervertebral discs. Presence of endplate defects as Schmorl’s Nodes and age (considering 18–60 yr range) did not show any significant influence on post contrast enhancement.

The protocol used in this work is a shorter version of the one developed by Rajasekaran et al [18]. We decided for practical reasons to study only the transport of contrast agent from blood flow to disc centre not including scans at 12 and 24 hours post injection. Furthermore, in the cited work a triple-dose injection of gadodiamide was used, whereas we chose to inject double-dose of gadoteridol. The latter contrast agent has the advantage to be more stable over time: the half life of gadodiamide is 35 s, the one of gadoteridol is 3 h [26]. We also chose to calculate the non-normalised difference between post and pre contrast images, in contrast with other works in which the difference was normalised with respect to the pre-contrast signal intensity [18], [27]. In our opinion this is unnecessary as it would introduce variability in the outcome not dependent on contrast medium concentration; on the other hand, it also means that results can only be presented as a difference in signal intensity, due to the lack of alternatives in normalising T1-weighted images. A preliminary attempt to use muscle tissue as reference was considered failed due to extremely high intra-rater variability, possibly due to the change in orientation of muscle fibres in different locations.

The profile of enhancement over time in all ROIs was studied only on disc with “normal”, “healthy” endplate. A smooth endplate allows for an easy segmentation of the disc space into “layers”, and guarantees the presence of reasonable symmetry on coronal and transversal planes. Analysing the transport in the disc without endplate defects, it is possible to appreciate the slow diffusion of the contrast medium from the bony endplate into the disc over time. The maximum concentration of contrast agent in the vascularised bone is just after injection and then decline as kidneys filter it out from the blood circulation. The EPZs show a general high enhancement at all time points, peaking at POST_2H. Arun at el [27] found similar results in their data regarding what they named “Peripheral region”, but is comparable to what we define as EPZ, with a peak after 3 hours from injection, and lack of a clear pattern. At POST_5MIN and POST_10MIN, the EPZs demonstrate lower enhancement than the bone, but since 2 hours after injection and thereafter, the concentration of contrast agent inside the disc is higher than in the blood stream. In accordance with data from Rajasekaran et al [18], the peak of enhancement in the central part of the disc is around 6 hours after injection: on average at this time points there is no gradient between Peripheral disc and Central disc. Data suggest that transport happens mostly in a symmetric fashion from the cranial and the caudal endplates.

Among the variables that influence post-contrast enhancement in Central Disc, disc height has the strongest (negative) association. This variable explains for 29% of data variability (at POST_4H) considering all discs. However, disc height among low to medium degenerated disc explains no more than 20% of data variability. These results are in accordance with results of Niinimaki et al [21], and was expected. Indeed, assuming a completely avascular disc, the contrast medium is dragged towards the disc centre mostly by a gradient of concentration, and the thicker the disc is, the longer is the distance between the vascularised endplate and the disc centre.

Transport depends also on degree of degeneration: a degenerated disc (Pfirrmann grading 4 and 5) presents a higher post contrast enhancement than a disc with low or medium degeneration (Pfirrmann grade 1, 2 and 3), but no significant difference is detectable at any time point between low and medium degenerated discs. If disc height is inserted in the regression model, Pfirrmann grading is still significantly associated with enhancement, indicating that this effect is not only due to the decrease disc height present in highly degenerated disc.

Degeneration was also evaluated with CSF- adjusted T2-weighted SI, which has been shown to be a highly repeatable measure that can be easily extracted from standard T2-weighted images [24], and estimates the mean disc water content compared to CSF in a continuous fashion. This index shows a significant negative correlation with age, but this correlation is weaker than in other studies [28]. A possible explanation for this is that the subjects involved in this study were all suffering of severe spinal problems, which often led to subsequent surgery and therefore hardly represent a random population. Also, with respect to other studies using this index, the numerosity of population is highly reduced, and this may have an influence on the resulting correlation. A significant correlation between CSF- adjusted T2-weighted SI and post-contrast enhancement is present if all the discs are considered, but when only low and medium degenerated discs are considered the correlation is not significant, despite a substantial difference of CSF T2-weighted SI among Pfirrmann grade 1, 2 and 3. If disc height is inserted in the model for all discs, the association with enhancement is only partially present (i.e., only at POST_4H) : CSF-adj T2-weighted SI discriminate better low and medium degeneration, but is less effective in discriminating between Pfirrmann grade 4 and 5, that are mainly distinguished by the difference in disc height. This data support similar findings of Niinimaki et al [21], but are partially in contrast with the results of Rajasekaran et al [18]. In the latter work, authors stated that “mild degeneration” is characterised by decreased enhancement, but it is not clear on which of the presented numerical results this statement was based. However, they also found an increase in enhancement in highly degenerated discs, that was explained as a sign of severe endplate damage, and possibly also of extensive vascularisation [20]. It should be noted, that if this is the case, it would be incorrect to refer to a mere “diffusion” process, as contrast agent would be transported into the disc by blood flow. Data presented in this work could be seen as corroborative of the results of Rodriguez et al [10], who studying cadaveric sample reported that total endplate mobility and porosity increase with degeneration.

Another factor influencing degeneration is the presence of MCs in adjacent endplates, which significantly increases post-contrast enhancement with respect to other discs. The presence of a higher enhancement at POST_10MIN suggests an increase of vascularisation in such discs, since virtually no contrast agent should penetrate into the central disc in such a short time only via diffusion from the endplate. If different types of MCs are considered separately, discs adjacent to type II and I/II MCs enhance significantly more than disc adjacent to type 0 endplates, while discs adjacent to MCs type I enhance similarly to type 0 discs. This may be coherent with the consideration that MCs I are in an earlier stage of degeneration. These findings are partially in contrast with data reported by Niinimaki et al [21]. The latter paper also found an increase of enhancement in disc adjacent to MCs, but MC I/II enhanced more than MC II. They argued that this effect could be a consequence of the higher vascularity in MC I. However, the number of MCs in their study was low, as only 9 MC I/II, 3 MC II and no pure MC I were present.

Another possible classification of the endplate is based on the presence of “defects”, usually regarded as “Schmorl’s nodes”. These were originally described as protrusion of disc tissue through the endplate into the vertebral marrow [29], but today they are often more broadly intended as any kind of endplate lesion [30].

Our results show that discs having at least an endplate defect do not significantly enhance more at the disc centre. Only endplates marked as “irregular”, which are adjacent only to heavily degenerated disc and are always associated with MC, do enhance significantly more than other discs with the same level of degeneration at POST_10MIN and POST_2H. The classification of “endplate irregularity” is not clearly recognised, though, but the significant early enhancement suggests that it could be a marker of a disc with extended vascularisation. Niinimaki et al found that the presence of endplate defects had a positive influence on post contrast enhancement [21]. Given the lack of a precise definition of endplate defect, the difference between our results may be due to different classification criteria.

Another relevant result is that age does not influence post-contrast enhancement, but is marginally correlated with CSF- adjusted T2-weighted signal intensity. This fails to confirm data from Rajasekaran et al [18] who stated that post contrast enhancement imaging could distinguish “aged disc” from “degenerated” ones. It should however be noted that in their work the studied population had a minimum age of 10y. In pre-adolescents, the endplate presents a zone of growth cartilage adjacent to the bone which is penetrated by cartilage canals facilitating nutrient transport into this area of the disc during growth [2]. Therefore, it is not surprising that immature discs enhance more than mature discs, but our results show that post contrast imaging fails to differentiate young adults from older adults.

There are several limitations to this research. One of the most important is the relative noise superimposed to the signal. In this setup MRI is effectively used as a sensor for the presence of contrast agent in a specific tissue voxel. High noise level is present in our data, but also in data shown in works using post-contrast imaging [18], [21]. Possible source of noise are various, i.e. MRI field inhomogeneities, movement artefacts, spatial shift between slices couples with the extremely heterogeneous disc biochemical characteristics, and Partial Volume Effects, which is the dilution of signal from small structures due to the limited spatial resolution, causing underestimation of signal amplitude and spillover into surrounding tissues [31].

Even though noise superimpose to the signal is significant, we believe that noise could have hidden significant effects from data, but it unlikely resulted in false positives statistical results. However, this suggest that it is not appropriate to evaluate the “time-intensity curve” on the single disc as it has been proposed by Rajasekaran et al [20], without a proper data reliability analysis to confirm that adequate technical counter-measures can maintain noise level into appropriate limits.

Another limitation is due to the use of a gadolinium-based contrast agent to infer the diffusion properties of disc cells nutrients, such as oxygen and glucose, due to their different physicochemical nature: gadolinium-based contrast agents have larger molecular weight and radius in comparison to the nutrients of the disc cells, and a slower diffusion should therefore be expected. Another limitation is the lack of precise knowledge on the evolution of the concentration of contrast agent in the blood flow over time. As the contrast agent dose increases linearly with subject's weight, as customary, but blood volume change also depending with gender, height, lean mass [32], the actual concentration at time zero is likely not the same in all patients. Also the concentration on time may have been influence by different renal clearance among subjects. These differences may have an influence on the final concentration of the contrast agent inside the disc, may the magnitude of this influence is not known.

In conclusion, disc height, high level of degeneration and presence of MCs are factors that increase post contrast enhancement in the centre of IVDs. Endplate close to degenerated disc and endplate affected by MCs may be more permeable to the contrast agent, but the increase in enhancement could be also due to the penetration of capillaries in degenerated discs. Presence of endplate defects does not show any significant influence on post contrast enhancement, but the lack of a clear classification of endplate defects as seen on MRIs may be shadowing some effects. In the considered population, the effect of age on post contrast enhancement could not be demonstrated.

## References

[1] White AA, Panjabi MM (1990) Clinical biomechanics of the spine. Philadelphia:Lippincott Williams & Wilkins. 752 p.

[2] Grunhagen T, Wilde G, Soukane DM, Shirazi-Adl A, Urban JP (2006) Nutrient supply and intervertebral disc metabolism. J Bone Joint Surg Am 88 Suppl 230–35. 16595440 10.2106/JBJS.E.01290

[3] Urban J, Holm S, Maroudas A, Nachemson A (1982) Nutrition of the intervertebral disc: Effect of fluid flow on solute transport. Clin Orthop Rel Res 170: 296–302. 7127960

[4] Galbusera F, Mietsch A, Schmidt H, Wilke H-J, Neidlinger-Wilke C (2013) Effect of intervertebral disc degeneration on disc cell viability: A numerical investigation. Comput Methods Biomech Biomed Engin 16(3): 328–337. 10.1080/10255842.2011.619184 21970697

[5] Shirazi-Adl A, Taheri M, Urban JPG (2010) Analysis of cell viability in intervertebral disc: Effect of endplate permeability on cell population. J Biomech 43(7): 1330–1336. 20167323 10.1016/j.jbiomech.2010.01.023

[6] Razaq S, Wilkins RJ, Urban JPG (2003) The effect of extracellular pH on matrix turnover by cells of the bovine nucleus pulposus. Eur Spine J 12(4): 341–349. 12883962 10.1007/s00586-003-0582-3 PMC3467790

[7] Bernick S, Cailliet R (1982) Vertebral end-plate changes with aging of human vertebrae. Spine 7(2): 97–102. 7089697 10.1097/00007632-198203000-00002

[8] Roberts S, Menage J, Eisenstein S (1993) The cartilage end-plate and intervertebral disc in scoliosis: Calcification and other sequelae. J Orthop Res 11(5): 747–757. 8410475 10.1002/jor.1100110517

[9] Benneker LM, Heini PF, Alini M, Anderson SE, Ito K (2005) 2004 young investigator award winner: Vertebral endplate marrow contact channel occlusions and intervertebral disc degeneration. Spine 30(2): 167–173. 15644751 10.1097/01.brs.0000150833.93248.09

[10] Rodriguez AG, Slichter CK, Acosta FL, Rodriguez-Soto AE, Burghardt AJ, et al (2011) Human disc nucleus properties and vertebral endplate permeability. Spine 36(7): 512–520. 21240044 10.1097/BRS.0b013e3181f72b94 PMC3062730

[11] Van der Veen A, van Dieen J, Nadort A, Stam B, Smit T (2007) Intervertebral disc recovery after dynamic or static loading in vitro: Is there a role for the endplate? J Biomech 40(10): 2230–2235. 17182043 10.1016/j.jbiomech.2006.10.018

[12] Holm S, Maroudas A, Urban JPG, Selstam G, Nachemson A (1981) Nutrition of the intervertebral disc: Solute transport and metabolism. Connect Tissue Res 8(2): 101–19. 6453689 10.3109/03008208109152130

[13] Bartels EM, Fairbank JCT, Winlove CP, Urban JPG (1998) Oxygen and lactate concentrations measured in vivo in the intervertebral discs of patients with scoliosis and back pain. Spine 23(1): 1–7. 9460145 10.1097/00007632-199801010-00001

[14] Urban MR, Fairbank JC, Etherington PJ, Loh FL, Winlove CP, et al (2001) Electrochemical measurement of transport into scoliotic intervertebral discs in vivo using nitrous oxide as a tracer. Spine 26(8): 984–990. 11317125 10.1097/00007632-200104150-00028

[15] Ibrahim MA, Haughton VM, Hyde JS (1995) Effect of disk maturation on diffusion of low-molecular-weight gadolinium complexes: An experimental study in rabbits. Am J Neuroradiol 16(6): 1307–1311. 7677031 PMC8337856

[16] Ibrahim M, Haughton V, Hyde J (1994) Enhancement of intervertebral disks with gadolinium complexes: Comparison of an ionic and a nonionic medium in an animal model. Am J Neuroradiol 15(10): 1907–1910. 7863940 PMC8334267

[17] Ibrahim M, Jesmanowicz A, Hyde J, Estkowski L, Haughton V (1994) Contrast enhancement of normal intervertebral disks: Time and dose dependence. Am J Neuroradiol 15(3): 419–423. 8197936 PMC8334299

[18] Rajasekaran S, Babu JN, Arun R, Armstrong BRW, Shetty AP, et al (2004) ISSLS prize winner: A study of diffusion in human lumbar discs: A serial magnetic resonance imaging study documenting the influence of the endplate on diffusion in normal and degenerate discs. Spine 29(23): 2654–2667. 15564914 10.1097/01.brs.0000148014.15210.64

[19] Rajasekaran S, Venkatadass K, Naresh Babu J, Ganesh K, Shetty AP (2008) Pharmacological enhancement of disc diffusion and differentiation of healthy, ageing and degenerated discs. Eur Spine J 17(5): 626–643. 18357472 10.1007/s00586-008-0645-6 PMC2367412

[20] Rajasekaran S, Vidyadhara S, Subbiah M, Kamath V, Karunanithi R, et al (2010) ISSLS prize winner: A study of effects of in vivo mechanical forces on human lumbar discs with scoliotic disc as a biological model: Results from serial postcontrast diffusion studies, histopathology and biochemical analysis of twenty-one human lumbar scoliotic discs. Spine 35(21) 1930: 1943. 10.1097/BRS.0b013e3181e9a156 20838264

[21] Niinimäki J, Korkiakoski A, Parviainen O, Haapea M, Kuisma M, et al (2009) Association of lumbar artery narrowing, degenerative changes in disc and endplate and apparent diffusion in disc on postcontrast enhancement of lumbar intervertebral disc. MAGMA 22(2): 101–109. 18949498 10.1007/s10334-008-0151-1

[22] Pfirrmann CWA, Metzdorf A, Zanetti M, Hodler J, Boos N (2001) Magnetic resonance classification of lumbar intervertebral disc degeneration. Spine 26(17): 1873–1878. 11568697 10.1097/00007632-200109010-00011

[23] Klein S, Staring M, Murphy K, Viergever MA, Pluim JPW (2010) Elastix: A toolbox for intensity-based medical image registration. IEEE Transactions on Medical Imaging 29(1): 196–205. 19923044 10.1109/TMI.2009.2035616

[24] Videman T, Gibbons LE, Battié MC (2008) Age-and pathology-specific measures of disc degeneration. Spine 33(25): 2781–2788. 19050585 10.1097/BRS.0b013e31817e1d11

[25] Battié MC, Videman T, Gibbons LE, Fisher LD, Manninen H, et al (1995) Determinants of lumbar disc degeneration: A study relating lifetime exposures and magnetic resonance imaging findings in identical twins. Spine 20(24): 2601–2612. 8747238

[26] Idée JM, Port M, Raynal I, Schaefer M, Le Greneur S, et al (2006) Clinical and biological consequences of transmetallation induced by contrast agents for magnetic resonance imaging: A review. Fund Clin Pharmacol 20(6): 563–576. 10.1111/j.1472-8206.2006.00447.x 17109649

[27] Arun R, Freeman BJC, Scammell BE, McNally DS, Cox E, et al (2009) 2009 ISSLS prize winner: What influence does sustained mechanical load have on diffusion in the human intervertebral disc?: An in vivo study using serial postcontrast magnetic resonance imaging. Spine 34(21): 2324–2337. 19755934 10.1097/BRS.0b013e3181b4df92

[28] Niemeläinen R, Videman T, Dhillon SS, Battié MC (2008) Quantitative measurement of intervertebral disc signal using MRI. Clin Radiol 63(3): 252–255. 18275864 10.1016/j.crad.2007.08.012

[29] Junghanns H, Schmorl G (1971) The human spine in health and disease. New York: Grune & Stratton. 504 p.

[30] Wang Y, Videman T, Battié MC (2012) Lumbar vertebral endplate lesions: Prevalence, classification, and association with age. Spine 37(17): 1432–1439. 22333959 10.1097/BRS.0b013e31824dd20a

[31] Boos N, Wallin A, Schmucker T, Aebi M, Boesch C (1994) Quantitative MR imaging of lumbar intervertebral discs and vertebral bodies: Methodology, reproducibility, and preliminary results. Magn Reson Imaging 12(4): 577–587. 8057762 10.1016/0730-725x(94)92452-x

[32] Holme S, Elfath MD, Heaton A, Whitley P, McNeil D (2008) Prediction of red cell and blood volumes distribution by various nomograms: do current nomograms overestimate? Transfusion 48(5): 910–916. 18208408 10.1111/j.1537-2995.2007.01619.x

